# Suicide attempts in LGBTQ+ youth in Switzerland: Qualitative insights in school-based risk factors

**DOI:** 10.1093/eurpub/ckac129.676

**Published:** 2022-10-25

**Authors:** NJ Bomolo, N Koschmieder, A Gavin, S Kupferschmid, C Bourquin, L Michaud, A Pfister

**Affiliations:** 1 Institute of Public Health, ZHAW Zurich University of Applied Sciences, Winterthur, Switzerland; 2 School of Social Work, Lucerne University of Applied Sciences and Arts, Lucerne, Switzerland; 3 Psychiatric Liaison Service, Lausanne University Hospital and University of Lausanne, Lausanne, Switzerland; 4 Integrated Psychiatry Winterthur, Winterthur, Switzerland

## Abstract

**Background:**

Studies show that the LGBTQ+ population is particularly vulnerable to suicidal thoughts and behavior. This vulnerability is even more pronounced in the younger population. However, in Switzerland, qualitative studies on this topic are missing. Our study investigates the processual dynamics and background of suicide attempts of LGBTQ+ youths while looking into their subjective meaning. Here, behaviors of help-seeking are also from interest. In addition, the burdens and resources associated with being LGBTQ+ are explored. By better understanding the process of suicide attempts, we can identify relevant contexts of the respondents’ experiences and illustrate how to enhance suicide prevention strategies. We are referring here to the school context.

**Methods:**

From 2021 until 2024, we interview LGBTQ+ youths in the German- and French-speaking parts of Switzerland who have tried to end their lives between the ages of 14 to 25 (max. three attempts). Applying a multi-perspective approach, we interview persons from their social environment if agreed. Recruitment is based on ‘theoretical sampling’. Data collection and analysis follow the grounded theory methodology. As of July 2022, the sample consists of 18 persons: 3 bisexual women, 1 lesbian woman, 2 gay men, 7 transgender persons, and 5 persons with fluid identities.

**Results:**

Through preliminary analysis, the school context could be identified as one relevant burdening context in the respondents’ experience and suicide attempt process. In this respect, respondents experienced complicated social relationships: e.g., bullying, social exclusion, and pressure to conform. Moreover, the school environment was experienced by some as LGBTQ+ hostile.

**Conclusions:**

Our current findings support the necessity to integrate schools as important stakeholders in suicide prevention but highlight a need for LGBTQ-specific and LGBTQ-sensitive orientations to suicide prevention strategies.

